# Research on anthracnose grade of *Camellia oleifera* based on the combined LIBS and THz technology

**DOI:** 10.1186/s13007-022-00883-1

**Published:** 2022-04-20

**Authors:** Li Bin, Wang Qiu, Zhan Chao-hui, Han Zhao-yang, Yin Hai, Liao Jun, Liu Yan-de

**Affiliations:** Institute of Optical-Electro-Mechatronics Technology and Application, East China Jiao Tong University, National and Local Joint Engineering Research Center of Fruit Intelligent Photoelectric Detection Technology and Equipment, Nanchang, 330013 China

**Keywords:** Joint spectroscopy, Laser-induced breakdown spectroscopy, Qualitative analysis, Terahertz

## Abstract

**Background:**

Anthracnose of *Camellia oleifera* is a very destructive disease that commonly occurs in the *Camellia oleifera* industry, which severely restricts the development of the *Camellia oleifera* industry. In the early stage of the *Camellia oleifera* suffering from anthracnose, only the diseased parts of the tree need to be repaired in time. With the aggravation of the disease, the diseased branches need to be eradicated, and severely diseased plants should be cut down in time. At present, aiming at the problems of complex experiments and low accuracy in detecting the degree of anthracnose of *Camellia oleifera*, a method is proposed to detect the degree of anthracnose of *Camellia oleifera* leaves by using terahertz spectroscopy (THz) combined with laser-induced breakdown spectroscopy (LIBS), so as to realize the rapid, efficient, non-destructive and high-precision determination of the degree of anthracnose of *Camellia oleifera*.

**Results:**

Mn, Ca, Ca II, Fe and other elements in the LIBS spectrum of healthy and infected *Camellia oleifera* leaves with different degrees of anthracnose are significantly different, and the Terahertz absorption spectra of healthy *Camellia oleifera* leaves, and *Camellia oleifera* leaves with different degrees of anthracnose there are also significant differences. Partial least squares discriminant analysis (PLS-DA), support vector machine (SVM), and linear discriminant analysis (LDA) are used to establish the fusion spectrum anthracnose classification model of *Camellia oleifera*. Among them, the Root mean square error of prediction (RMSEP) and the prediction determination coefficient R^2^p of THz-LIBS-CARS-PLS-DA of prediction set are 0.110 and 0.995 respectively, and the misjudgment rate is 1.03%; The accuracy of the modeling set of THz (CARS)-LIBS (CARS)-SVM is 100%, and the accuracy of prediction set is 100%, after preprocessing of the multivariate scattering correction (MSC), the accuracy of the THz-LIBS-MSC-CARS modeling set is 100%, and the accuracy of prediction set is 100%; The accuracy rate of THz-LIBS-MSC-CARS-LDA of modeling set is 98.98%, and the accuracy rate of the prediction set is 96.87%.

**Conclusion:**

The experimental results show that: the SVM model has higher qualitative analysis accuracy and is more stable than the PLS-DA and LDA models. The results showed that: the THz spectrum combined with the LIBS spectrum could be used to separate healthy *Camellia oleifera* leaves from various grades of anthracnose *Camellia oleifera* leaves non-destructively, quickly and accurately.

## Background

*Camellia oleifera* is known as the four largest woody oil plants globally. It mainly grows in the south of China; it is a kind of pure natural high-grade oil peculiar to our country and has good economic benefits. In recent years, the plantation area of *Camellia oleifera* has continued to expand, and the *Camellia oleifera* industry has also driven the local economy. However, with the expansion of the planting area, the diseases and insect pests of *Camellia oleifera* have become more and more serious, which not only affects the development of the *Camellia oleifera* industry and the economic benefits of the planting land but also poses new problems for the prevention and elimination of diseases and insect pests of the *Camellia oleifera*. *Camellia* anthracnose is the primary disease of *Camellia oleifera*, and the disease is extremely destructive. In severe cases, it can cause the *Camellia oleifera* to lose flowers and fruits, dry branches and die, and finally, the whole plant decays; it is mainly common in Hunan, Jiangxi, Guangdong, and other provinces that are in the central distribution area of *Camellia* in China [1]. In the early stage of the disease, the diseased parts of the tree should be repaired in time. As the condition worsens, the diseased branches of the tree should be eradicated in time. The seriously diseased trees must be cut down in time.

At present, there are two major aspects to detect plant diseases [2]. One is laboratory testing techniques, Shuai Xiao-chun [3], etc. through tissue separation method to isolate and purify pathogens at the junction of disease and health, and seven typical anthracnose fungi were identified by morphological methods; P. Parikka et al. [4] used conventional polymerase chain reaction (PCR) technology to detect early spore anthracnose of strawberry tissue; Liu Yan-de et al. [5] used flame atomic absorption spectrometry (FAAS) to detect the degree of anthracnose of *Camellia oleifera*. Although these detection techniques have high precision, the experimental processing process is very cumbersome, and many requirements are required for the experimenters, which will cause secondary pollution. The other aspect is the spectrum detection, and image texture detection, such as Wu Nan [6], etc. analyzed the visible-near infrared spectrum characteristics of the *Camellia* canopy after anthracnose infection by BP neural network model, and the anthracnose of the *Camellia* leaf is successfully detected; Wang Xian-feng et al. [7] performed image processing on the images of diseased leaves, and cucumber leaf downy mildew, brown spot, and anthracnose were successfully identified by the statistical analysis system (SAS). Although spectral detection and image detection are simpler and faster than laboratory detection, however, it is seldom detected in the grade of plant disease. Therefore, it is necessary to find a fast, efficient, simple, and high-precision detection method to detect plant diseases.

Laser-induced breakdown spectroscopy (LIBS) is an elemental analysis technology based on atomic emission spectroscopy and laser-plasma emission spectroscopy. The LIBS experimental method is simple, and it is a fast, direct, and multi-element analysis technology. In recent years, it has been widely used in plant element analysis [8]. Wang et al. [9] used laser-induced breakdown spectroscopy(LIBS) combined with discrimination analysis (DA) technology to successfully identify six types of tea; Denilson M et al. [10] used LIBS technology to detect trace and macro-element of vegetables; Zhao Shang-yong et al. [11] detected six different ginseng by LIBS and successfully distinguished six types of ginseng. These studies mainly use LIBS to detect the properties of elements to identify and classify samples, which prove that LIBS can identify and classify samples according to different element contents in samples. This paper mainly studies the classification accuracy of different grades of *Camellia oleifera* anthracnose. After *Camellia oleifera* is diseased, the contents of nutrient elements such as Fe and Mn will change. According to the spectral changes detected by LIBS, the changes of nutrients inside the leaves are determined, finally, the LIBS spectral data are used to model the determination of *Camellia oleifera* anthracnose grades. LIBS technology can detect plant elements but cannot detect macromolecular substances, while Terahertz (THz) technology can detect macromolecular substances. The THz spectrum refers to electromagnetic waves with a frequency between 0.1 and 10THz. It has the dual characteristics of microwave and infrared. Due to the weak interaction between most organic macromolecules in the matter, skeleton vibration, dipole rotation, and vibration transition frequency correspond to the Terahertz spectrum, which makes the Terahertz technology has great potential in the application of food adulteration detection [12, 13]. Li et al. [14] used THz spectral technology to identify green tea from four different origins. Liu Yan-de et al. [15] analyzed the Terahertz spectrum of purple rice and dyed purple rice in the range of 0.5–2.5THz through terahertz spectrum technology, and purple rice and dyed purple rice were distinguished. Terahertz detection is mainly based on the characteristics of the fingerprint spectrum to identify the chemical components in the samples to classify the samples, which is in line with the direction and purpose of this research. Therefore, this research decided to use terahertz technology to detect the level of *Camellia* anthracnose.

Aiming at the current methods for detecting the degree of anthracnose of *Camellia oleifera* have disadvantages, such as complexity, low efficiency, environmental pollution, and low accuracy. As the complementary of LIBS and THz, in order to further improve the detection accuracy of *Camellia* anthracnose, the combination of LIBS and THz with chemometric methods is proposed to achieve non-destructive, fast, efficient, and high-precision detection the degree of anthracnose of *Camellia oleifera* in the paper.

## Methods

### Sample preparation

The experimental samples used in this study are healthy *Camellia oleifera* leaves and anthracnose of *Camellia oleifera* leaves picked in the *Camellia oleifera* planting area in Nanchang, Jiangxi. The *Camellia oleifera* leaves were classified and pretreated by morphological, and PCR techniques and used as subsequent experimental samples. Five different types of *Camellia oleifera* leaves are picked, respectively. There are 110, 100, 110, 110, and 170 samples of mild, mild to moderate, moderate, and severe anthracnose of *Camellia oleifera* samples and healthy *Camellia oleifera* leaves, respectively. It is composed of anthracnose of *Camellia oleifera* leaves with different proportions of the black-brown diseased area to the total leaf area. Among them, leaves with anthracnose lesion areas less than 1/4 on *Camellia* leaves are called mild anthracnose oil-tea leaves. Those with lesion area less than 1/2 and greater than 1/4 are called mild-to-moderate anthracnose oil-tea leaves. The lesion area is greater than 1/2 less than 3/4 are called moderately oleifera leaves, and those with more than 3/4 of the diseased area are called severe oleifera leaves. The processing process of the experimental samples: picking, washing (using deionized water), sorting, drying (60 °C for 6 h), grinding, sieving (200 mesh sieve), tableting (10Mpa pressure for 1 min), bagging, labeling, use LIBS and THz instruments to detect samples.

### Processing method

#### *Collection of* LIBS *spectra*

In this experiment, the nutrient elements in the leaves of *Camellia oleifera* are detected using the LIBS instrument of Ocean Optics Company's model MX2500 + . The solid-state laser—Nd: YAG laser (Quantel, Big Sky Laser Ultra50) is used to generate 1060 nm light excitation. The instrument contains 5 Channels. The intensity of the LIBS spectrum is affected by the distance between the focusing lens and the sample and the delay time. Therefore, the LIBS equipment parameters are set and optimized accordingly. Through the comparison and analysis of LIBS spectral signals, the optimal parameter settings of the LIBS equipment are: single laser trigger, laser energy set to 50 mJ, and the wavelength range of the spectrometer is 198.71 nm ~ 727.69 nm, the optical resolution is 0.1 nm, the integration time is 1 μs; the distance between the focusing lens and the sample surface is set to 4.1 cm, and the delay time is set to 2.5 μs. Each sample collects 8 LIBS spectral data values dispersedly; the purpose is to reduce the error and reduce the influence of the uneven distribution of elements in the leaves of *Camellia oleifera*.

**Fig. 1:**
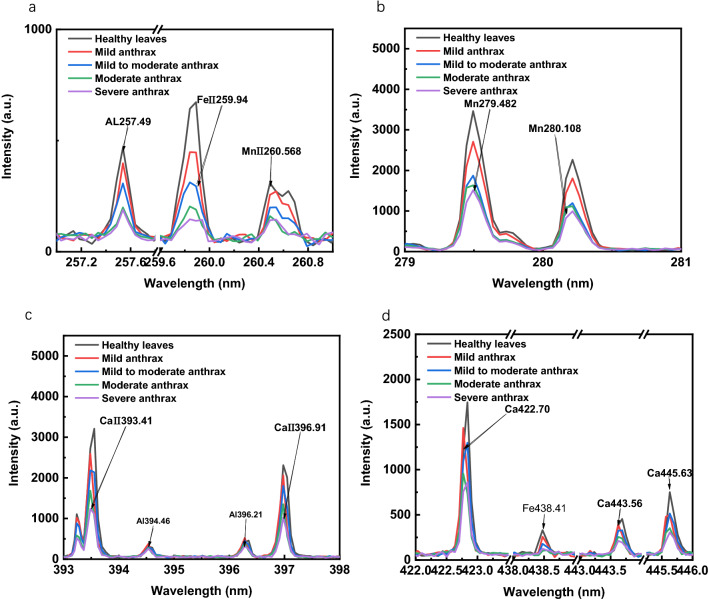
Comparison of characteristic peaks of elements in LIBS257-447 nm Camellia oleifera leaves, **a** 257–262 nm, **b** 279–281 nm, **c** 393–398 nm, **d** 422–446 nm, **a**–**d** represent the elements in the camellia samples detected by LIBS in the 257–446 nm spectral segment

The spectral line data obtained from the experiment correspond to the National Institute of Standards and Technology (NIST) database, and the elements are calibrated within the error range. The elements detected by the LIBS instrument in this experiment are shown in Fig. 1. Figure 1a is the elements detected by LIBS within 257 ~ 262 nm, which are Al257.49, FeII259.94, MnII260.568; and Fig. 1b is the element information detected in the 279 ~ 281 nm spectral band, Mn279.482, and Mn280.108; Fig. 1c is the element information detected in the 393 ~ 398 nm spectral band, CaII393.41, and CaII396.91; Fig. 1d are elements detected in the 422 ~ 446 nm spectral range, including four characteristic spectral lines: Ca422.70, Fe438.41, Ca443.56, and Ca445.63. It can be seen from Fig. 1 that the characteristics peak intensity of healthy oil-tea *Camellia* leaves is higher than that of diseased oil-tea *Camellia* leaves, and the four characteristic spectral lines of diseased oil-tea *Camellia* leaves gradually decrease with the increase of the disease level. The main reason is that these elements are all necessary elements for the growth of *Camellia oleifera*. As the degree of anthracnose on the leaves of *Camellia oleifera* increases, the content of these types of elements gradually decreases.

#### THz spectrum collection

The Terahertz Time-Domain Spectroscopy (THz-TDS) system used in this experiment is a terahertz system developed by Advantest, Japan. The model is TAS7400. The spectrum measurement is carried out in the time-domain transmission mode. The spectrum collection range of the system is 0.5–7 THz, the resolution is 7.6 GHz, the laser center wavelength is 1560 nm, and the laser power is 400 μW. Because moisture significantly influences the terahertz spectrum, the spectrum collection process is carried out in a closed box, and dry air is continuously pumped to make the air humidity of the measurement environment below 10%. The temperature is controlled at about 25 °C. In order to reduce the error, each sample is measured at three points, and each point is measured twice.

**Fig. 2:**
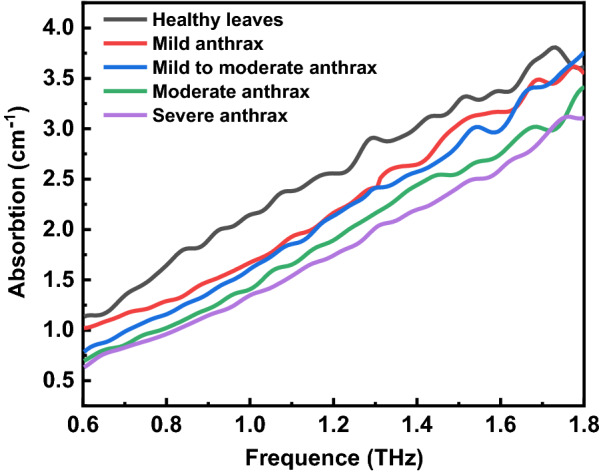
Spectra of 0.6–1.8THz absorption coefficients

Due to the THz absorption coefficient spectrum, the spectrum higher than 1.8THz has obvious noise. This may be due to the low signal-to-noise ratio in the high-frequency area due to the scattering effect. The part of the spectrum below 0.6THz that is less than 0 and the noise part should also be intercepted. Therefore, the absorption coefficient spectrum of 0.6 ~ 1.8THz is taken for analysis. Figure 2 shows the THz absorption coefficient spectra of five samples after an interception. As the frequency increases, the absorption coefficient of the sample also increases. Due to the fingerprint spectrum characteristics of the terahertz spectrum, it can be seen from the figure that the absorption spectra of healthy leaves are significantly different from those of diseased leaves, and the absorption intensity gradually decreases as the diseased grade of *Camellia oleifera* increases.

### Data processing and analysis

#### Principal component analysis

Principal component analysis (PCA) is a multivariate statistical method [16]. While preserving the original variable information as much as possible, the basic idea is to transform the original high-dimensional data into a low-dimensional feature variable of linearly independent through an orthogonal transformation. The transformed variables are called principal components (PCs). PCA is a linear algorithm and cannot explain the complex polynomial relationships between features [17, 18]. Under normal circumstances, when the cumulative variance contribution rate of the current n PCs is large enough (generally 85%), the original data can be replaced with the first n PCs. The principal component analysis process is as follows [19]:

1. Standardize the original spectral data $X_{i}$, and then calculate the covariance matrix ${\mathbf{S}}$.
  1 $$X_{{\text{i}}}^{*} = \frac{{X_{i} - mean(X_{i} )}}{{std(X_{i} )}}(i = 1,2,3, \ldots, n)$$
  2 $${\mathbf{S}} = \frac{{X^{*T}X^{*}} }{{n-1}}$$
  where i is the i-th sample and n is the number of samples.
2. Calculate the eigenvalues and correlation coefficient matrix ${\mathbf{R}}$ of the slope variance matrix ${\mathbf{S}}.$
  3 $${\mathbf{R}} = (r_{ij} )_{m \times m}$$
  4 $$r_{ij} = \frac{{\sum\limits_{k = 1}^{n} {X_{ki}^{*} } X_{kj}^{*} }}{n - 1},({\text{i}},{\text{j}} = 1,2,3,m)$$
  Among them: $r_{ii} = 1$,$r_{ij} = r_{ji}$,$r_{ij}$ is the correlation coefficient between the sample $i$ and the variable $j$, ${\text{m}}$ is the number of eigenvalues, and k is the k-th standardized spectral data. Then calculate the eigenvalues and sort them in descending order, λ_1_ ≥ λ_2_ ≥ λ_3_ ≥ … ≥ λ_m_ ≥ 0.
3. According to the cumulative variance contribution rate, the appropriate number of principal components is selected and the model is established.

#### PCA-LDA analysis

Linear discriminant analysis (LDA) is a supervised classification method. The basic idea of LDA classification is to extract the best identifiable low-dimensional features from high-dimensional features and then use these selected features to classify samples. Make the samples of the same kind cluster together as much as possible, while the samples of the different kinds are separated as much as possible; that is, the between-class variance is the largest, and the intra-class variance is the smallest [20, 21] since LDA uses the Fisher criterion function, LDA is also called Fisher linear Discriminant Analysis (FDA) [22]. The Fisher criterion function is

5 $$j(W) = \arg \mathop {\max }\limits_{W} \frac{{\left| {W^{T} S_{b} W} \right|}}{{\left| {W^{T} S_{w} W} \right|}}$$

$W$ is the projection direction, ${\mathbf{S}}_{{\mathbf{b}}}$ is the inter-class dispersion matrix, and ${\mathbf{S}}_{{\mathbf{w}}}$ is the intra-class dispersion matrix. And are defined as formula (6) and formula (7), respectively.

6 $${\mathbf{S}}_{{\mathbf{b}}} = \sum\limits_{i = 1}^{C} {N_{i} } (\mu_{i} - \mu )(\mu_{i} - \mu )^{T}$$

7 $${\mathbf{S}}_{{\mathbf{w}}} = \sum\limits_{i = 1}^{C} {\sum\limits_{{x_{i}^{(k)} \in X_{i} }} {\left( {x_{i}^{(k)} - \mu_{i} } \right)} } \left( {x_{i}^{(k)} - \mu_{i} } \right)^{T}$$

$C$ is the number of sample categories, $N_{i} (i = 1,2,...,C)$ is the number of class $i$ samples, $\mu = \frac{1}{N}\sum\limits_{j = 1}^{N} {x_{j} }$ is the mean vector of all samples, $N$ is the total number of samples, $x_{j}$ is the $j$ sample vector,$X_{i}$ is category $i$ samples, $x_{i}^{(k)}$ is the $k$ sample vector of class $i$, and $\mu_{i} = \frac{1}{{N_{i} }}\sum\limits_{{x_{i}^{(k)} \in X_{i} }} {x_{i}^{(k)} }$ is the mean vector of class $i$ samples.

LDA requires that the input matrix X cannot be too many; otherwise, it cannot be run. For example, the X input of the SVM is 110 × 787, and there are 787 spectral variables. However, LDA cannot accommodate 787 spectral variables, so it is usually PCA-LDA. X [110 × 787] is compressed into T [110 × 20] principal component score variables through PCA, and we select several score variables as input, to ensure the correct operation of LDA.

#### Support vector machine analysis

The support vector machine (SVM) algorithm is a supervised learning model. Its main idea is to find the optimal separation hyperplane and use a nonlinear mapping function to map the training data set to the high-dimensional space to maximize the distance between different class samples [23]. SVM has a good generalization ability in the classification of different types of samples [17]. In the process of establishing the SVM model, determining the penalty factor c and the kernel parameter g is the key to establishing the SVM model [24]. The parameters in this paper are obtained by a grid search to get the optimal c and g, as shown in Table 1. In the grid, it can be seen that the optimal Validation accuracy is 99.3%. Choose one of 99.3% to get the optimal training accuracy of 100%, and the corresponding optimal c is 0.01 and g is 1.

**Table 1:** Grid search determines parameters

Accuracy%	c	0.01	0.1	1	10	100
Gamma		1	2	3	4	5
0.01	1	99.1	99.1	98.3	99.1	99.1
0.1	2	98.6	98.6	99.1	98.1	99.1
1	3	**99.3**	99.1	99.1	99.3	98.3
10	4	99.1	99.3	99.3	98.6	99.3
100	5	98.6	98.3	99.3	99.3	99.1

	Start	End	Levels	SVs		
Log10(Gamma)	− 2	2	5	Validation	Accuracy	99.3%
Log10(c)/Nu	− 2	2	5	Training	Accuracy	100%
				C		0.01
				Gamma	Value	1

#### Extract feature variables

The specific number of feature variables of various spectra extracted by CARS,UVE is shown in Table 2.

**Table 2:** Extract feature variables

Spectrum						
Variable	LIBS	THz	THz-LIBS-CARS	THz-LIBS-UVE	THz(CARS)-LIBS(CARS)	THz(UVE)-LIBS(UVE)
CARS	460	4	708	–	4 + 460	–
UVE	195	52	–	378	–	52 + 195

## Results

### Partial least squares discriminant modeling analysis of LIBS, THz and LIBS-THz based on CARS and UVE

A total of 392 *Camellia oleifera* leaf samples are detected by THz, which are divided into 295 modeling samples according to the classification of 3:1 by K-S, including 50, 45, 50, 51, and 99 samples of mild anthracnose, mild to moderate anthracnose, moderate anthracnose, severe anthracnose, and healthy *Camellia oleifera* leaf samples. There are 97 samples in the prediction set, including 17, 14, 16, 17, and 33 leaves of mild, mild to moderate, moderate, severe, and healthy *Camellia oleifera*. A total of 600 LIBS spectral sample points are used to detect anthracnose of *Camellia* oil leaves. K-S is divided into model set 409 and prediction set 191 according to 3:1. Among the modeling sets, the leaf samples of mild anthracnose, mild to moderate anthracnose, moderate anthracnose, severe anthracnose, and healthy *Camellia oleifera* are 110, 100, 110, 110 and 170, respectively. And in the prediction set, the number of mild anthracnose leaf samples, mild to moderate anthracnose leaf samples, moderate anthracnose leaf samples, severe anthracnose leaf samples, and healthy *Camellia oleifera* leaf samples are 27, 25, 27, 44, 68, respectively, as shown in Table 3.

**Table 3:** K-S classification results of THz and LIBS detection

Grade of anthracnose	THZ detection			LIBS detection		
	Modeling set/piece	Prediction set/piece	Total/piece	Modeling set/piece	Prediction set/piece	Total/piece
Mild	50	17	67	83	27	110
Mild to moderate	45	14	59	75	25	100
Moderate	50	16	66	83	27	110
Severe	51	17	68	66	44	110
Healthy	99	33	132	102	68	170

It can be seen from Table 4 that the PLS-DA model established by THz spectroscopy to detect the anthracnose of *Camellia oleifera* has a misjudgment rate of modeling set and prediction set are 56.12% and 60.20%. The LIBS spectrum establishes a PLS-DA model to detect anthracnose of *Camellia oleifera*. Although the misjudgment rate of the prediction set is 16.23%, the misjudgment rate of the modeling set reached 31.54%. When the LIBS and THz spectra are spliced, the LIBS-THz-PLS-DA modeling error rate is 29.49%, which is lower than the modeling set error rate of the THz and LIBS models established separately, so LIBS-THz is proved to be able to improve the accuracy of identifying the grade of anthracnose. Since the results of establishing the PLS-DA model after THz is extracted by CARS and UVE features are abysmal, the data is not used as a reference. After the CARS feature extraction, the misjudgment rate of the modeling set of the PLS-DA model for LIBS is 0.49%, and the misjudgment rate of the prediction set is 0. However, the misjudgment rate of the modeling set after UVE feature extraction is 5.38%, and the misjudgment rate of the prediction set is 21.98%, indicating that the LIBS detection of the degree anthracnose of *Camellia oleifera* is better with CARS to extract feature values. Perform CARS and UVE feature extraction on the spectra directly spliced between LIBS and THz, and then perform PLS-DA modeling, respectively. From Table 4, it can be seen that the calibration standard deviation of LIBS-THz-CARS is RMSEC = 0.103, and the calibration determination coefficient R^2^c = 0.995, modeling set misjudgment rate is 0, the prediction standard deviation RMSEP = 0.110, the prediction determination coefficient R^2^p = 0.995, the misjudgment rate of the prediction set is 1.03%; the LIBS-THz-UVE's RMSEC = 0.180, R^2^c = 0.985, the modeling set misjudgment rate is 1.02%, RMSEP = 0.405, R^2^p = 0.927, and the misjudgment rate of the prediction set is 23.71%. It once again proves that CARS feature extraction is effective in detecting the anthracnose grade of *Camellia oleifera* leaves. Next, the LIBS spectrum and the THz spectrum are extracted with CARS and UVE features, respectively, and then the spectrum is spliced, and the intermediate fusion is performed. The modeling effect is still better after the CARS feature extraction. The misjudgment rate of the modeling set is 1.02%, and the prediction set misjudgment rate is 0. After UVE feature extraction, the misjudgment rate of the modeling set is 5.10%, and the misjudgment rate of the prediction set is 18.37%.

**Table 4:** PLS-DA modeling of single spectrum and fusion spectrum

Spectrum	Modeling set			Prediction set		
	RMSEC	R^2^c	Misjudgment rate	RMSEP	R^2^p	Misjudgment rate
THz	0.861	0.684	56.12%	0.775	0.661	60.20%
LIBS	0.515	0.876	31.54%	0.331	0.947	16.23%
LIBS-CARS	**0.176**	**0.986**	**0.49%**	**0.130**	**0.992**	**0**
LIBS-UVE	0.249	0.971	5.38%	0.436	0.908	21.98%
THz-LIBS	0.528	0.874	29.49%	0.436	0.914	4.12%
THZ-LIBS-CARS	**0.103**	**0.995**	**0**	**0.110**	**0.995**	**1.03%**
THZ-LIBS-UVE	0.180	0.985	1.02%	0.405	0.927	23.71%
THZ(CARS)-LIBS(CARS)	**0.188**	**0.985**	**1.02%**	**0.160**	**0.985**	**0**
THZ(UVE)-LIBS(UVE)	0.252	0.973	5.10%	0.382	0.918	18.37%

The five types of samples are represented by 0, 1, 2, 3, 4. 0–1, 1–2, 2–3, 3–4, and 4–5 respectively represent the classification range of the five types of samples, they are considered as a classified mistake if they exceed the classification range.

### Support vector machine modeling of LIBS, THz and LIBS-THz based on CARS and UVE

In establishing the SVM model, the grid search method is adopted to select the optimal SVM parameters c and g, and the model is verified by cross-validation. Finally, part of the prediction set samples that do not participate in the modeling are reserved for external validation of the model. In order to obtain the optimal model, this paper imported the spectra pretreated by MSC, baseline correction, and normalization into the SVM algorithm and established the model with the two most commonly used kernel functions of SVM, and compared the model results under different pretreatments and different kernel functions. Finally, it is determined that the modeling set and prediction set established by the Linear kernel preprocess by MSC have the highest accuracy. Table 5 shows the accuracy of the modeling set and prediction set of SVM based on CARS and UVE for LIBS, THz, and LIBS-THz. It can be seen from Table 5 that the accuracy of the modeling set and modeling set pretreated by MSC is basically 100%. Combined with the accuracy of the prediction set, it can be seen that the accuracy of the modeling set of THz (CARS)-LIBS(CARS)-SVM is 100%, and the accuracy of the prediction set is 100%. After MSC preprocessing, the accuracy of the modeling set of THz-LIBS-MSC-CARS-SVM is 100%, and the accuracy of the prediction set is 100%. That is, these two models are the best results of SVM modeling.

**Table 5:** SVM modeling of single spectrum and fusion spectrum

Spectrum	Modeling set	MSC-modeling set	Prediction set	MSC-prediction set
THz	82.65%	74.49%	61.85%	53.06%
LIBS	100%	100%	94.37%	95.78%
LIBS-CARS	**100%**	**100%**	**97.36%**	**96.31%**
LIBS-UVE	100%	100%	94.73%	95.78%
THz-LIBS	100%	100%	90.63%	95.83%
THz-LIBS-CARS	**100%**	**100%**	**95.83%**	**100%**
THz-LIBS-UVE	100%	100%	95.83%	96.90%
THz(CARS)-LIBS(CARS)	**100%**	**100%**	**100%**	**97.79%**
THz(UVE)-LIBS(UVE)	100%	99.66%	97.94%	97.94%

**Fig. 3:**
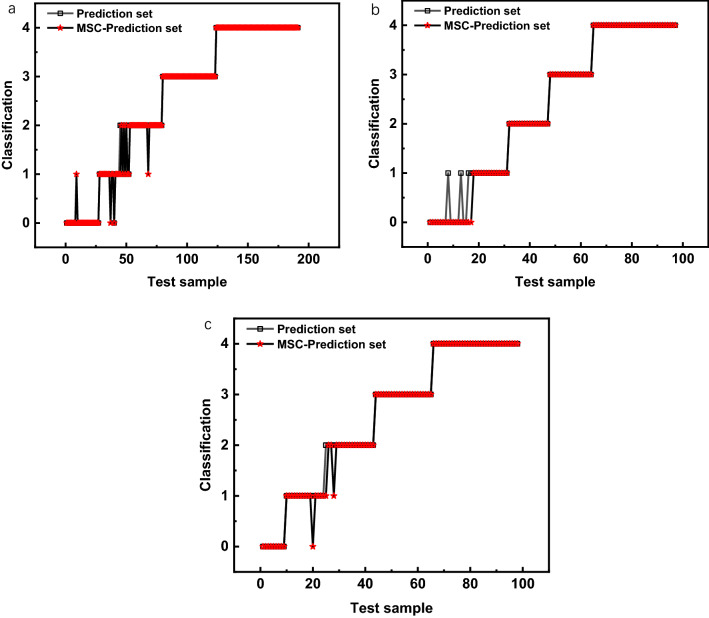
Comparison of the accuracy of SVM prediction set and MSC preprocessing prediction set, **a** LIBS-CARS prediction set and MSC-prediction set, **b** THz-LIBS-CARS prediction set and MSC- prediction set, **c** THz (CARS)-LIBS(CARS) prediction set and MSC-prediction set

Figure 3 is a comparison diagram of the prediction set accuracy of LIBS-CARS, THz-LIBS-CARS, THz (CARS)-LIBS(CARS) without preprocess and the SVM model with MSC preprocess. Figure 3a the prediction set without preprocessing has one sample with mild to moderate anthracnose of *Camellia oleifera* misclassified as mild anthracnose of *Camellia oleifera*, and four samples with mild to moderate anthracnose of *Camellia oleifera* are wrongly classified as moderate anthracnose. A total of five samples are misclassified; the prediction set after MSC pretreatment included one sample that classified the mild anthracnose of *Camellia oleifera* into mild to moderate anthracnose, and two samples of mild to moderate anthracnose of *Camellia oleifera* are misclassified as mild, the three samples with mild to moderate anthracnose of *Camellia oleifera* are wrongly classified as moderate anthracnose of *Camellia oleifera*, and one sample with moderate anthracnose of *Camellia oleifera* is wrongly classified as mild to moderate anthracnose of *Camellia oleifera*, a total of seven misclassifications. Figure 3b in the prediction set without pretreatment, four samples of mild anthracnose of *Camellia oleifera* are misclassified into mild to moderate anthracnose of *Camellia oleifera*, and four samples are misclassified. The accuracy of the prediction set after MSC preprocessing is 100%, without misclassification. Figure 3c the accuracy of the prediction set without pre-processing is 100%. After the MSC pre-processing, the prediction set is incorrectly classified into three types: One sample with mild to moderate anthracnose of *Camellia oleifera* is incorrectly classified into mild and two samples with moderate anthracnose of *Camellia oleifera* is incorrectly classified into mild to moderate anthracnose of *Camellia oleifera*. In summary, the modeling accuracy and prediction accuracy of the THz-LIBS-MSC-CARS-SVM model is 100%, and the modeling accuracy and prediction accuracy of THz (CARS) -LIBS (CARS)-SVM model is 100%, these two models are the best in the SVM model for detecting anthracnose on *Camellia* leaves.

### LIBS, THz, LIBS-THz linear discriminant analysis modeling based on CARS and UVE

**Fig. 4:**
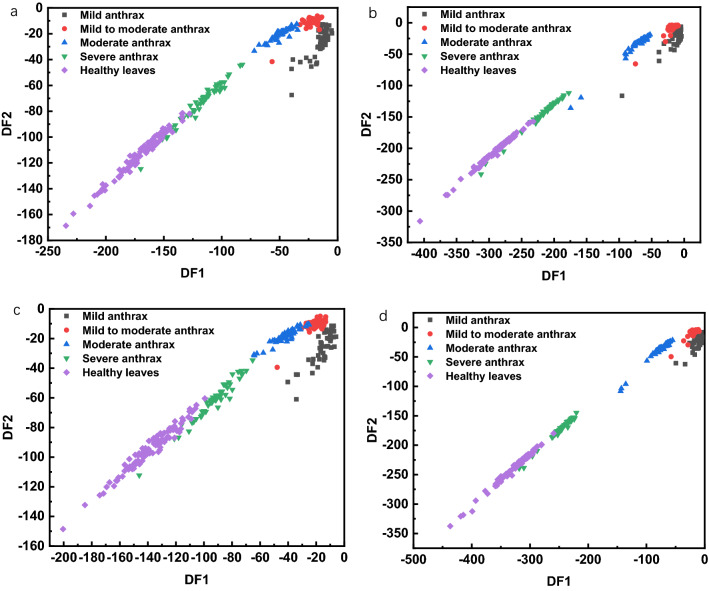
Plane classification diagram of linear discriminant analysis model of modeling set samples, **a** THz-LIBS-CARS, **b** THz-LIBS-MSC-CARS, **c** THz-LIBS-UVE, **d** THz-LIBS-MSC-UVE

Enter the variables to establish the LDA model. After the model-based, import the reserved part of the prediction set samples into the established LDA classification model to evaluate the model. As shown in Fig. 4, the plane classification diagram is drawn by the first two discriminant functions of the modeling set samples. In the two spectra selected from the nine spectra and the LDA model preprocessed by MSC, the distribution of samples of different types of modeling sets has obvious classification boundaries. Because the LDA classification diagram mainly represents the degree of aggregation of samples of the same type, the distribution of samples of different types does not affect the classification accuracy of the model.

From Fig. 4a and b, it can be seen that the accuracy of the THz-LIBS-CARS modeling set is increased by 0.34% after MSC preprocessing, the classification boundary (b) is more precise than (a), the classification clustering degree (b) is higher than (a), combined with Table 6, the accuracy of the prediction set is increased by 4.17%; from (c) and (d), it can be seen that although the accuracy of the modeling set of THz-LIBS-UVE has not changed after MSC preprocessing, it can be seen from the figure. The classification boundary (d) is more precise than (c), and the classification clustering degree (d) is higher than (c). Combined with Table 6, the prediction set accuracy of THz-LIBS-UVE is improved by 9.28% after the MSC preprocessing, which is of great significance to improving classification accuracy.

**Table 6:** LDA modeling of single spectrum and fusion spectrum

Spectrum	Modeling set	Prediction set	MSC-modeling set	MSC-prediction set
THz	98.30%	61.85%	–	–
LIBS	87.29%	83.68%	90.22%	93.15%
LIBS-CARS	89.98%	83.68%	92.91%	96.31%
LIBS-UVE	96.82%	81.15%	94.38%	93.19%
THz-LIBS	91.53%	85.42%	97.63%	93.75%
THz-LIBS-CARS	**98.64%**	**92.70%**	**98.98%**	**96.87%**
THz-LIBS-UVE	98.98%	82.47%	98.98%	91.75%
THz(CARS)-LIBS(CARS)	89.46%	87.62%	93.54%	97.94%
THz(UVE)-LIBS(UVE)	94.22%	84.53%	95.58%	95.876%

It can be seen from Table 6 that the accuracy of the THz modeling set is 98.3%, but the accuracy of the prediction set is 61.85%, which is prone to under-fitting. The accuracy of the modeling set from LIBS-LDA to LIBS-MSC-LDA increased from 87.29 to 90.22%, and the accuracy of the prediction set rose from 83.68 to 93.15%. The accuracy of the modeling set in LIBS-UVE-LDA is 96.82%, but the accuracy of the prediction set is only 81.15%, the model is not very stable, but after MSC preprocessing, the accuracy of the modeling set is 94.38%, and the prediction set accuracy is 93.19%, which is relatively close, and the model is relatively stable.

Table 7 compares the results of the optimal models in PLS-DA, SVM, and LDA. It can be seen that the best models are THz (CARS)-LIBS(CARS)-SVM and THz-LIBS-MSC-CARS-SVM, the accuracy of the modeling set of the two models is 100%, and the accuracy of the prediction set is also 100%. It is the model with the best stability and highest accuracy among all models.

**Table 7:** Comparison of PLS-DA, SVM, LDA model results

Spectrum	Modeling set		Prediction set	
	Classification accuracy	Misjudgment rate	Classification accuracy	Misjudgment rate
THz-LIBS-CARS-PLS-DA	100%	0	98.97%	1.03%
THz(CARS)-LIBA(CARS)-SVM	100%	0	100%	0
THz-LIBS-MSC-CARS-SVM	100%	0	100%	0
THz-LIBS-MSC-CARS-LDA	98.98%	1.02%	96.87%	3.13%

## Discussion

**Fig. 5:**
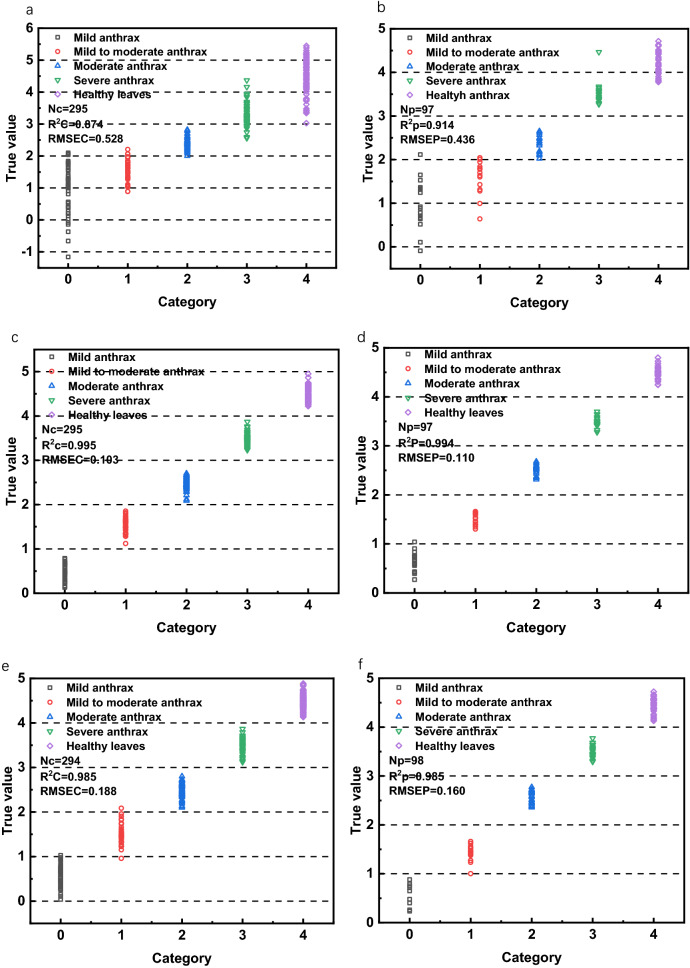
PLS-DA classification diagram of Camellia oleifera leaves detected by different spectra, **a** LIBS-THz modeling set, **b** LIBS-THz prediction set, **c** LIBS-THz-CARS modeling set, **d** LIBS-THz-CARS prediction set, **e** THz(CARS)-LIBS(CARS) modeling set, **f** THz(CARS)-LIBS(CARS) prediction set

From Fig. 5a, b, it can be seen that the misjudgment rate of LIBS-THz-PLS-DA is relatively high, with 87 misjudgments in the modeling set and 22 misjudgments in the prediction set; after CARS feature extraction, Fig. 5c, d the false-positive rate of LIBS-THz-CARS-PLS-DA is significantly reduced, the modeling set hasn’t misjudgments, and the prediction set has one misjudgment. Figure 5e, f the misjudgment rate was lower than that of the LIBS single spectrum after CARS feature extraction, two misjudgments in the modeling set and don’t have a misjudgment in the prediction set. Mainly because Competitive Adaptive Reweighted Sampling (CARS) is a feature variable selection method that combines Monte Carlo sampling and PLS model regression coefficients, imitating the principle of "survival of the fittest" in Darwin's theory. It is utilized to filter the variables in the spectrum that contribute more to the spectrum. According to the comparison of misjudgment rates in Fig. 5a–f, it is necessary to combine LIBS and THz to detect the low-level fusion of anthrax of *Camellia oleifera* for feature extraction. After feature extraction of LIBS and THz, respectively, and then spectral splicing (Fig. 5e, f), although the results are worse than those of low-level fusion LIBS-THz-CARS-PLS-DA, they are much better than those of direct spectral splicing LIBS-THz-PLS-DA, proving that intermediate fusion is meaningful. Although the result of intermediate fusion in this paper is worse than that of low-level fusion, there may still be some other feature extraction and model building methods to make the result of intermediate fusion better than that of low-level fusion, which is still worth trying in the future research. To sum up, the best result obtained in the establishment of the PLS-DA model is LIBS-THz-CARS-PLS-DA; that is, the PLS-DA model is established after the splicing of LIBS and THz spectra through the extraction of CARS features.

From Table 5, a separate comparison of the prediction set without preprocessing and the prediction set with MSC preprocessing shows that except for LIBS-CARS, the results of other models are preferable to the original model after MSC preprocessing. Therefore, MSC preprocessing is necessary to establish a SVM model for detecting the degree of anthrax of *Camellia oleifera*. Since multivariate scatter correction is used to correct the offset effect in the spectral data, the particle size of the sample is not uniform during the sample preparation process, and the scattering benefit is prone to occur during the spectral acquisition process, which can be eliminated by MSC. It can be seen from Table 6, from LIBS to LIBS-CARS to LIBS-MSC-CARS; the accuracy of the modeling set and prediction set has been improved, indicating that MSC preprocessing and CARS feature extraction is necessary for the LDA model of anthracnose detection of *Camellia oleifera* leaves. Uninformative variable elimination (UVE) is a feature extraction method based on PLS model regression coefficient stability analysis, which is mainly developed to eliminate variables that have no valid information in the original spectral data. From Table 6, although the model after UVE feature extraction has higher modeling set accuracy, the prediction set accuracy is not high, and the model stability is poor. In the LDA model for detecting anthrax on *Camellia oleifera* leaves, the variables extracted from the CARS feature are more suitable for this model than those extracted from UVE feature. Comprehensive classification accuracy and modeling accuracy show that the accuracy is the highest, and the model with the best stability is THz-LIBS-MSC-CARS.

## Conclusions

In this paper, the combined THz and LIBS with chemometric methods are used to detect the degree of anthracnose of *Camellia oleifera*. The non-destructive and accurate determination of the degree of anthracnose of *Camellia oleifera* is achieved. Firstly, the models of PLS-DA are established, according to the model’s results, the THz-LIBS-CARS-PLS-DA is the best result in all PLS-DA models, it’s RMSEC and R^2^c are 0.103 and 0.995, respectively, and the misjudgment rate is 0; The RMSEP and R^2^p of it are 0.110 and 0.995, respectively, and the misjudgment rate is 1.03%. Then, the models of SVM are established, the THz (CARS)-LIBS(CARS)-SVM and THz-LIBS-MSC-CARS-SVM are the best, the accuracy of modeling set of them are 100%, and the accuracy of prediction set of them are 100%. Finally, the models of LDA are established, the THz-LIBS-MSC-CARS-LDA is the best model, the accuracy of the modeling set is 98.98%, and the accuracy of the prediction set is 96.87%. The research results show the SVM has the highest accuracy, prediction accuracy, and best stability. Therefore, combined THz and LIBS with the SVM model can realize non-destructive, fast, and high-precision detection on the degree of anthracnose of *Camellia oleifera*. This study provides an experimental reference for the detection of anthracnose of *Camellia oleifera.*

## Data Availability

Not applicable.

## Abbreviations

THz: Terahertz
LIBS: Laser-induced breakdown spectroscopy
PLS-DA: Partial least squares discriminant analysis
SVM: Support vector machine
LDA: Linear discriminant analysis
RMSEP: Root mean square error of prediction
RMSEC: Root mean square error of calibration
MSC: Multivariate scattering correction
PCA: Principal component analysis
CARS: Competitive adaptive reweighted sampling
UVE: Uninformative variable elimination
